# Development of the Bullying and Health Experiences Scale

**DOI:** 10.2196/ijmr.1835

**Published:** 2012-11-09

**Authors:** Tanya Beran, Lauren Stanton, Ross Hetherington, Faye Mishna, Shaheen Shariff

**Affiliations:** 1 University of CalgaryDepartment of Community Health SciencesCalgary, ABCanada; 2 Catholic District School Board of Eastern OntarioAncaster, ONCanada; 3 University of TorontoDepartments of Pediatrics and Public Health SciencesToronto, ONCanada; 4 University of TorontoFactor-Inwentash Faculty of Social WorkToronto, ONCanada; 5 McGill UniversityDepartment of Integrated StudiesMontreal, QCCanada

**Keywords:** mental health, school bullying, cyberbullying, peer victimization, psychosocial impact, children

## Abstract

**Background:**

Until recently, researchers have studied forms of bullying separately. For 40 years, research has looked at the traditional forms of bullying, including physical (eg, hitting), verbal (eg, threats), and social (eg, exclusion). Attention focused on cyberbullying in the early 2000s. Although accumulating research suggests that bullying has multiple negative effects for children who are targeted, these effects excluded cyberbullying from the definition of bullying.

**Objective:**

This paper responds to the need for a multidimensional measure of the impact of various forms of bullying. We used a comprehensive definition of bullying, which includes all of its forms, to identify children who had been targeted or who had participated in bullying. We then examined various ways in which they were impacted.

**Methods:**

We used an online method to administer 37 impact items to 377 (277 female, 100 male) children and youth, to develop and test the Bullying and Health Experience Scale.

**Results:**

A principal components analysis of the bullying impact items with varimax rotation resulted in 8 factors with eigenvalues greater than one, explaining 68.0% of the variance. These scales include risk, relationships, anger, physical injury, drug use, anxiety, self-esteem, and eating problems, which represent many of the cognitive, psychological, and behavioral consequences of bullying. The Cronbach alpha coefficients for the 8 scales range from .73 to .90, indicating good inter-item consistency. Comparisons between the groups showed that children involved in bullying had significantly higher negative outcomes on all scales than children not involved in bullying.

**Conclusions:**

The high Cronbach alpha values indicate that the 8 impact scales provide reliable scores. In addition, comparisons between the groups indicate that the 8 scales provide accurate scores, with more negative outcomes reported by children involved in bullying compared to those who are not involved in bullying. This evidence of reliability and validity indicates that these scales are useful for research and clinical purposes to measure the multidimensional experiences of children who bully and are bullied.

## Introduction

Encountering bullying behavior is one of the most distressing experiences for children and adolescents, particularly when such behaviors occur over a prolonged period of time [1,2]. Bullying behavior is strongly associated with a number of detrimental cognitive, psychological, health, and behavioral outcomes that can persist into adulthood [3,4]. Although researchers have studied bullying since the 1970s [5], few measures of children’s functioning related to bullying exist. Considering that its newest form, cyberbullying, occurs electronically, it seems reasonable to consider developing a bullying and health experiences scale that could be administered electronically. The purpose of this study was to develop, pilot, and evaluate an online, multidimensional instrument to assess the health experiences of children who are bullied and/or bully others.

### Health Experiences Associated with Bullying

A substantial body of research supports specific deleterious correlates of bullying [6]. In terms of cognitive functioning, victims of bullying report lower school attachment and lower academic achievement compared to children not involved in bullying [7,8].

Researchers have also documented psychological and health factors related to bullying. Findings from a national study that examined 123,227 children 11, 13, and 15 years of age residing in 28 countries found bullying was strongly related to psychological symptoms, including nervousness, sleep difficulty, and feelings of rejection, loneliness, and helplessness [9]. Other studies have found that frequent exposure to bullying is significantly related to internalizing disorders, with signs and symptoms of anxiety, depression, social withdrawal, diminished self-esteem or diminished sense of self-worth, and suicide ideation [1,10,11]. Fekkes [10] extended previous research inquiries into the relationship between mental health symptoms and bullying using longitudinal data. Findings suggest that children who were regularly bullied at the beginning of the school year were more likely to develop new mental health and health-related symptoms throughout the year, including depression, anxiety, bed-wetting, abdominal pain, and tension [10]. Similar to findings related to traditional bullying, children who are bullied online are significantly more likely to experience psychological symptoms than children not involved in bullying [12].

In addition to these cognitive and psychological symptoms, many researchers have demonstrated a number of behavioral problems related to bullying. Similar to victims of traditional bullying, victims of cyberbullying are likely to display externalizing behaviors, such as drug and alcohol use [12]. In addition, they are more likely to report carrying a weapon to school compared to children who have not experienced online bullying [12]. Other studies have found that frequent exposure to bullying experiences is linked with higher reports of eating disorders, such as bulimia nervosa, among female youth [13].

Although they may be disliked by the peers they target, aggressive children experience average levels of popularity [5]. Additionally, they do not necessarily exhibit low levels of self-esteem [14], are not highly anxious [5,14], nor are they highly depressed [14]. Nevertheless, the negative effects of bullying are not restricted to victimized children. Research has also documented negative health experiences for children who perpetrate the bullying. For example, they tend to have cognitive difficulties such as poor academic achievement [15,16]. They also tend to require mental health services [17]. They may exhibit conduct problems, such as vandalism [18], smoking [16], and drinking [16,18]. In addition, Holmes and Brandenburg-Ayres [19] reported that early bullying experiences significantly predicted later gang membership and incarceration.

In addition to children who bully and those who are victimized, there exists a group of children who both bully and are victimized (bully/victims). Hanish and Guerra [20] described these children as having the most disturbed functioning and note that they “are more likely to have emotional, behavioral, social, academic, and family problems.” Children who both bully others and are bullied are considered the most vulnerable to negative developmental outcomes [21].

Despite years of research, measures typically assess one or two of the cognitive, psychological, or behavioral dimensions discussed. The purpose of our research, therefore, is to create and test a multidimensional measure of the health experiences associated with bullying.

### The Internet as a Method of Data Collection

The Internet is an expedient means of transferring information and is recognized as a valid method of collecting qualitative and quantitative data [22,23]. It is also becoming an effective means of delivering general and personal information about health [24,25]. It is superior to traditional paper-based survey methods because it allows greater access to respondents through mass distribution and eliminates data entry errors arising from transcribing responses, which are entered into a database [26]. In addition, online data collection yields higher rates of completion than mail-out surveys [27]. Although it is not possible to verify the identity of the respondent, the validity of data from any method of data collection is always in question. Recall bias, misinterpretation of questions, fabrication of information, and so on are inherent to all data collection methods. It may be advantageous to use electronic methods with certain populations about certain topics of study. Given that youths ages 12-17 years use the Internet more than any other age group [28], they are likely to have access to and skill in using it for a variety of purposes. Therefore, it seems appropriate to use the Internet to ask youths questions about their behaviors while on the Internet. Using the Internet as a means of data collection is likely to provide valid and generalizable results about youths’ Internet experiences [29].

Power and dominance exerted through aggression are endemic in human societies. Although researchers have investigated this phenomenon for decades, multidimensional measures of children’s functioning related to bullying have not yet been developed. The purpose of our study was to comprehensively examine the health experiences of all forms of bullying using a web-based survey, and to determine whether it yields reliable and valid information when compared across groups of children who are bullied, bully others, neither, or both. We predict that children who are bullied and/or bully others are more likely to experience problems across cognitive, psychological, and behavioral domains of functioning than children who are not bullied.

## Methods

### Sample

We planned to recruit a convenience sample of 200 children with an open online survey.  We selected the Kids Help Phone website for recruitment because of its widespread use among youth [30]. Based on the average of 15,000 unique visits to the Kids Help Phone home page per week, and a response rate of 1%, we estimated it would take two weeks to recruit our sample. We actually recruited 377 subjects in two weeks. The completion rate was 100%. The sample included 377 children (*n* = 277 girls, *n* = 100 boys) from age 10 to 17 years (*M* = 13.90, *SD* = 1.84). Most children lived in urban (*n* = 307) compared to rural areas (*n* = 70); were born in Canada (*n* = 319) compared to outside of Canada (*n* = 58, all children were living in Canada at the time of the survey); and spoke English at home (*n* = 280) compared to another language (*n* = 97).

### Procedure

The Kids Help Phone website has been in existence since 1996 and provides professional counseling and referrals to people aged 5 to 20 years on any problem or concern. Through word-of-mouth and advertising, many children in Canada have become aware of this resource. Participants were recruited by placing a click-through badge with a message about the survey on the webpage. Visitors were shown a distorted password that they typed in. This password served as a security check to prevent automated programs from accessing the questionnaire. In cases where someone responded twice, we included only the first response.

### The Bullying and Health Experiences Scale

Respondents first read the following definition:

There are lots of ways to hurt someone. A person who bullies wants to hurt the other person. A person who bullies does it because they can. They may be older, stronger, bigger, or have other students on their side.

Then respondents were provided with examples of forms of bullying, as shown in Table 1.

**Table 1:** Forms of bullying and examples provided to respondents.

Form of Bullying	Examples
Physical	Hitting, kicking, or spitting
Verbal	Name calling, mocking, humiliating, or hurtful teasing
Social	Leaving someone out, gossiping, or spreading rumors
Electronic	Bullying on Facebook, MSN, email, or text messaging
Racial	Saying hurtful things about someone whose skin is a different color
Sexual	Kissing, hugging, grabbing, pinching, and saying something sexual
Sexual preference	Teasing someone for being gay whether they are or not

Children indicated whether and how often they experienced any of the listed forms of bullying within the last month using a Likert scale with anchors from “no” to “several times a week.” A similar question was administered about bullying others.

Children were then administered 37 items asking about their cognitive, psychological, and behavioral experiences. These items were obtained from a review of the research and various measures of children’s functioning. These were rated on a Likert scale according to their frequency using the following anchors: “never,” “only once or twice,” “two or three times a month,” “about once a week,” and “several times a week.” All items included a “no” response option. The questions constituted 8 subscales, as shown in Table 2.

**Table 2:** Subscales for rating behavioral experiences survey.

Subscale	Number of questions	Example
Positive relationships towards peers.	6	“Other students are kind and helpful to me.”
Anger	6	“I felt angry.”
Anxiety	7	“I worry a lot.”
Self-esteem	3	“I like myself.”
Risk behaviors	5	“I have been in a physical fight.”
Physical injury caused by being bullied	3	“I was hurt by someone at school, enough to need bandages or a doctor.”
Eating problems	4	“I threw up because I was upset.”
Drug use	3	“I have used drugs.”

## Results

For the questions measuring bullying victimization and perpetration, the Cronbach alphas were .77 and .71, respectively, indicating good internal consistency. To summarize, 31.3% of children reported being victimized in some way at least 2-3 times in the past month, and 11.1% reported bullying others at least 2-3 times in the past month. This is consistent with previous research [31]. Pearson product moment correlation coefficients among types of bullying perpetrated (r = 0.06 to 0.64, Mean = 0.28) and experienced (r = 0.016 to 0.54, Mean = 0.33) were low, suggesting that there was little overlap across different forms of bullying.

We analyzed the factor structure of children’s reports of health experiences. The Bartlett’s test of sphericity (Chi-square = 9346.06, *P* < .001) and the Kaiser-Meyer-Olkin measure of sampling adequacy (0.88) indicated that the sample was sufficient to evaluate the correlations and detect factors. The items were all subjected to principal components analysis with varimax rotation. The reliability was then determined by examining the internal consistency of the items that loaded under each factor. These analyses resulted in an 8-factor solution that explained 68.0% of the variance (see Table 3). These factors have eigenvalues greater than 1 and are named *anger* (Cronbach alpha = .87, 6 items), *relationships* (Cronbach alpha = .86, 6 items), *physical injury* (Cronbach alpha = .90, 3 items), *risk* (Cronbach alpha = .84, 5 items), *anxiety* (Cronbach alpha = .82, 7 items), *self-esteem* (Cronbach alpha = .92, 3 items), *drug use* (Cronbach alpha = .86, 3 items), and *eating problems* (Cronbach alpha = .73, 4 items). A few of the items had split loadings; however, reliability analyses indicated that they were consistent with other items within the factor, as shown in the table. When an item had a loading of greater than 0.40 on two components, the higher loading was used to assign it to a component [32].

**Table 3:** Types of health experiences determined from principal components analysis

	Anger	Relation-ships	Physicalinjury	Risk	Anxiety	Self-esteem	Drug use	Eating problems
I like myself.	.049	-.114	.023	-.010	.087	.901	.028	.028
I am able to do things as well as most other people.	.016	-.048	.064	.017	.069	.910	.005	.138
I have lots of good qualities.	.034	-.077	.013	.054	.087	.919	.060	.101
I like school.	-.306	.545	.040	-.119	.055	.067	-.335	-.108
I feel alone or left out at school.	-.060	.587	-.092	.035	-.308	-.243	.071	-.103
Other students are kind and helpful to me.	-.154	.773	-.117	-.084	-.038	.064	-.159	-.117
Other students like hanging out with me.	-.124	.844	-.113	.032	-.132	-.049	-.037	-.044
Other students accept me for who I am.	-.164	.793	-.110	-.040	-.128	-.040	-.056	-.154
I make friends easily.	-.084	.791	-.023	-.117	-.154	-.142	.127	-.033
I worry a lot.	.310	-.285	-.077	-.134	.592	.082	.155	.257
I worry about my family.	.124	-.099	.010	.001	.719	.013	.007	.034
I worry about doing well in school.	-.013	.057	.044	-.092	.778	.034	-.027	-.180
I worry about making friends.	.113	-.444	.054	.112	.560	.169	-.090	-.014
I worry about the future.	.015	-.185	.014	.079	.751	.039	.026	.124
My worries kept me up at night.	.399	-.182	.035	-.022	.519	.194	.031	.428
I have trouble catching my breath.	.292	-.195	.166	-.081	.358	.149	.165	.433
I was in a physical fight.	.338	-.060	.412	.546	.052	-.009	.147	-.037
I was in a physical fight where the other person needed bandages or a doctor.	.204	.026	.507	.621	-.039	.046	.263	.036
I was in trouble with the law.	.098	-.048	.228	.680	-.079	.072	.291	.255
I took weapons to school.	.085	-.084	.116	.834	-.023	.018	.134	.164
I damaged property.	.275	-.095	.136	.598	.051	-.037	.253	.083
I was hurt by someone at school, enough to need bandages or a doctor.	.067	-.121	.831	.160	.061	.021	.073	.210
I was hurt by someone while going to and from school, enough to need bandages or a doctor.	.034	-.146	.855	.233	-.011	.072	.114	.141
I was hurt by someone in my neighborhood, enough to need bandages or a doctor.	.089	-.080	.862	.170	.078	.012	.120	.139
I smoked cigarettes.	.181	-.073	.156	.153	.038	.073	.833	.070
I drank alcohol.	.165	.020	.107	.360	.048	-.018	.704	.268
I have used drugs.	.091	-.057	.144	.313	-.008	.044	.812	.156
I felt angry.	.697	-.277	-.049	.027	.235	-.027	.071	.152
I yelled/screamed/stomped my feet when angry.	.795	-.104	.045	.059	.062	.030	.010	.172
I swore at others when angry.	.737	-.039	.027	.120	.085	-.007	.180	.183
I broke things when angry.	.727	-.076	.158	.244	.044	.045	.165	.073
I hurt someone when angry.	.614	-.140	.230	.393	-.069	.039	.047	.046
I got angry more easily with others.	.748	-.217	.055	.087	.145	.063	.071	-.002
I threw up because I was upset.	.209	-.093	.237	.169	-.033	.052	.381	.613
I could not eat because I was so upset.	.191	-.196	.072	.104	.128	.150	.122	.663
I used medicine to help me lose weight.	.080	-.027	.313	.333	-.062	.083	.083	.622
I over ate to the point where I became sick.	.153	-.263	.349	.222	.035	.042	.101	.433

We compared the scores on these 8 dimensions across several profiles, including bully, victim, non-bully/victim, and bully/victim. First, we classified children as bullies if they reported bullying others 2-3 times per month or more, *and* being bullied no more than 1-2 times per month. Similarly, we coded children as victims if they reported being bullied 2-3 times per month or more, *and* bullying others not more than 1-2 times per month. Children who bullied and were bullied 2-3 times per month or more were often coded as bully/victims. We considered children who bullied and were bullied no more than 1-2 times per month as non-bully/victims. This classification system is typically used in the research [33].

The number of children in each group is shown in Table 4. The mean scores were calculated by taking the sum of all the items that loaded under each factor and dividing by the number of items for each factor. Thus, the possible range of mean values in Table 4 is from 1 to 5. Partial η^2^ provided effect size estimates in analyses of variance (ANOVAs) and were interpreted using Cohen’s [34] criteria (.01 = small, .09 = medium, .25 = large). Accordingly, there was a small difference for risk, anger, physical injury, anxiety, and eating problems across the bully classifications. Also, there was a moderate to large difference in relationship experiences. Self-esteem and drug use, however, did not significantly differ across bully or victim groups.

**Table 4:** Mean scores of health experiences subscales for bullies, victims, bully/victims, and non-bully/victims.

	Bully(*n* = 19)	Victim(*n* = 95)	Bully/Victim(*n* = 23)	Non-bully/Victim(*n* = 240)	F* *df* (3, 373)	*Partial* *Eta* ^*2*^
Risk	1.58	1.64	1.61	1.29	6.98	.05
Relationships	3.50	2.67	2.98	3.68	27.38	.18
Anger	3.46	3.29	3.54	2.68	8.83	.07
Physical injury	1.07	1.46	1.49	1.14	6.95	.05
Anxiety	3.56	3.78	3.88	3.30	7.56	.06
Self-esteem	3.33	3.25	3.29	3.17	0.18	.00
Eating problems	1.88	2.11	2.23	1.59	10.29	.08
Drug use	1.53	1.86	1.58	1.63	1.08	.01

*All *F* values are significant at *P* < .001 with the exception of self-esteem and drug use.

Post hoc analyses using Tukey’s HSD showed several significant differences (*P* < .001). Victimized children engaged in more risk behaviors, experienced fewer positive relationships and more physical injury, and reported higher levels of anger, anxiety, and eating problems than did children not involved in bullying. Children who admitted to bullying behaviors were more likely to report anger than were non-bully/victims. Bully/victims were more likely to report anger, anxiety, eating problems, and poor peer relationships compared to non-bully/victims. Finally, non-bully/victims were the least likely to report negative experiences.

## Discussion

Based on an extensive review of the literature, we created a 37-item multidimensional scale. Through web-based administration of these items, and analyses of factorial structure and reliability, we obtained evidence of their usefulness when assessing children’s health experiences. The subscales measure risk behaviors, relationship experiences, anger, physical injury, anxiety, drug use, self-esteem, and eating problems. These areas address many of the cognitive, psychological, and behavioral problems of children involved in bullying, as identified in previous research. The reliability coefficients are adequate and scores were significantly elevated for children who were victimized and/or perpetrated bullying.

### Bullying Roles

Significant differences in health experiences emerged among groups according to the type of involvement in bullying. Specifically, children who reported victimization also reported engaging in risk behaviors, such as weapons possession. This is consistent with findings published by Ybarra [12]. Children may be exposed to these risk behaviors through acts of bullying, or they may participate in these risk behaviors as a coping reaction to being bullied. Participation in these risky behaviors probably increases the likelihood of physical injury, as was found in our study. Children who participated in risky behaviors also reported fewer positive relationships with peers, which is consistent with research showing that children who are bullied may experience interpersonal victimization and social skills difficulties [12]. Several studies have substantiated that children who are bullied experience high levels of anger and anxiety [1,9,10,11]. Other health problems, such as eating problems involving vomiting or limiting food intake, were more often reported by children who were bullied compared to children not involved in bullying. This finding is also substantiated by previous research [13].

 Similar to children who were bullied, children who perpetrated bullying reported elevated levels of anger, as shown in previous research [35]. They did not report high levels of anxiety, which is also consistent with other studies [14]. In addition, they reported experiencing positive relationships, which supports research suggesting that bullies perceive themselves as liked by their peer group [5]. Indeed, bullying attracts attention from the peer group, which bullies may interpret as positive attention. Bullies are, moreover, likely to report similar levels of self-esteem as non-bully/victims [14], which is consistent with our findings. Contrary to our expectations, children who reported bullying others did not report high levels of risk behaviors. The absence of reported risky behaviors in this group could be related to the relatively young age of our sample. For example, a multinational study of the prevalence of substance use indicates that peak onset of alcohol and cannabis use is in mid- to late adolescence, and at age 18 for all other drugs [36].

We also included children who were both victims and perpetrators of bullying. Several health concerns were higher for these children than those not involved in bullying. That is, the bully/victim was likely to experience poor peer relationships, anger, anxiety, and eating problems, as suggested by previous research [37]. We found, however, that these children did not report the most negative outcomes, as expected. Rather, victims did. This could be due to the relatively strong self-esteem and positive relationships that the bully/victim reported, which may buffer negative outcomes. Moreover, children who are bullied and victimized typically receive the lowest levels of social support [38], which may explain the negative outcomes documented in most research. In our study, however, they may have been receiving support from the Kids Help Phone website. This support may have lessened the detrimental correlates of bullying, resulting in lower reporting of these negative behaviors.

### Significance and Implications

We chose not to use items about academic achievement and educational experiences because they did not appear to reliably or accurately measure the constructs of our scale. In addition, we did not include questions about depression. We recommend further research in item development on questions pertaining to academic achievement and depression. Moreover, considering that no single source could feasibly assess all aspects of functioning, we recommend that additional instruments be administered to provide a more comprehensive profile, particularly when designing or providing interventions or support. In addition, identifying children as bullies or victims based on experiencing any or all types of bullying created heterogeneous groups. However, given the large number of groups that would have been formed for each type and combinations of types of bullying, it was not possible to compare functioning across all groups. Generalizability of the results may be limited because participants were recruited from the Kids Help Phone website, which they may have been visiting because they were already troubled or seeking support. Although the prevalence of bullying we report is similar to other research, our participants may be unique on other unknown dimensions. Participants filled out a questionnaire via a computer using the Internet. Accordingly, this study was restricted to children who have access to a computer and the Internet. Because the survey was administered online, participants could not ask for clarification or help from the individual administering the questionnaire, which could have affected the accuracy of some responses. Finally, in common with all anonymous online research, it was not possible to verify the information provided by participants.

Despite these limitations, this study provides strong evidence for the usefulness of this multidimensional web-based survey on health experiences related to bullying. It further adds to the growing evidence of the detrimental impact of bullying among multiple profiles of children and youth, whether in the role of bully, victim, or bully/victim.
